# Study of the Chronology of Expression of Ten Extracellular Matrix Molecules during the Myogenesis in Cattle to Better Understand Sensory Properties of Meat

**DOI:** 10.3390/foods8030097

**Published:** 2019-03-13

**Authors:** Anne Listrat, Mohammed Gagaoua, Brigitte Picard

**Affiliations:** Université Clermont Auvergne, INRA, VetAgro Sup, UMR Herbivores, F-63122 Saint-Genès-Champanelle, France; mohammed.gagaoua@inra.fr or gmber2001@yahoo.fr (M.G.); brigitte.picard@inra.fr (B.P.)

**Keywords:** skeletal muscle, fetus, bovine, extracellular matrix, immunohistology

## Abstract

The sensory properties of beef are known to depend on muscle fiber and intramuscular connective tissue composition (IMCT). IMCT is composed of collagens, proteoglycans and glycoproteins. The differentiation of muscle fibers has been extensively studied but there is scarcity in the data concerning IMCT differentiation. In order to be able to control muscle differentiation to improve beef quality, it is essential to understand the ontogenesis of IMCT molecules. Therefore, in this study, we investigated the chronology of appearance of 10 IMCT molecules in bovine *Semitendinosus* muscle using immunohistology technique at five key stages of myogenesis. Since 60 days *post-conception* (dpc), the whole molecules were present, but did not have their final location. It seems that they reach it at around 210 dpc. Then, the findings emphasized that since 210 dpc, the stage at which the differentiation of muscle fibers is almost complete, the differentiation of IMCT is almost completed. These data suggested that for the best controlling of the muscular differentiation to improve beef sensory quality, it would be necessary to intervene very early (before the IMCT constituents have acquired their definitive localization and the muscle fibers have finished differentiating), i.e., at the beginning of the first third of gestation.

## 1. Introduction

Beef tenderness is one of the most important quality attributes for the consumer. It is often inconsistent and affects consumer satisfaction. The variability in final meat quality is due to several factors such as the differences in muscle characteristics [1], namely to the proportion of different types of muscle fibers [2,3,4] and the characteristics of intramuscular connective tissue (IMCT) [5,6]. Among the characteristics of IMCT, the total collagen and its solubility are studied more. IMCT is a composite network that can both withstand and transmit forces generated by muscle contraction. This complex network not only ensures the structural integrity of muscle, but also mediates the development and physiological behavior of muscle cells. IMCT maintains the structural integrity of muscle fibers by two layers, the endomysium that surrounds individual skeletal muscle fibers and the perimysium that bundles groups of muscle fiber. IMCT consists of cells and an ExtraCellular Matrix (ECM) that primarily consists of a composite network of fibrillar collagens wrapped in a matrix of proteoglycans (PGs). These later form large complexes by binding to other PGs and to fibrous proteins such as collagens. The PGs consist of a core protein linked by covalent bonds to several glycosaminoglycan chains (GAGs). The chemical structure of these GAGs varies between the different PGs. The PGs of the skeletal muscle are mainly chondroitin sulfate (CS). Among them, decorin, a small PG and versican, a large PG, have been associated with myogenesis [7]. 

The fibrillary collagens, more commonly known as total collagen (particularly type I that is the most abundant fibrillar collagen of the skeletal muscle) interact with tenascin-X, a glycoprotein, and type XII and XIV collagens, two non-fibrillar minor collagen types [8,9]. Collagen XII and XIV interact with decorin [10,11,12]). In vitro, the supposed role of tenascin-X and of collagen XII and XIV is to modulate the flexibility of the ECM and consequently, its mechanical properties [13,14]. From these properties and according to Bailey and Light [15], it has been suggested that these molecules could contribute to the meat texture. However, there is a scarcity in studies that addressed this question unless one work from our groups that studied the relation between decorin, tenascin-X, collagen XII and XIV and beef sensory properties [16]. According to our previous study, it seems important to study all these components together, in combination with total and insoluble collagen, as they would have complementary information and an additive role that would play on the final texture quality of meat.

It is worthwhile to note that the differentiation of muscle fiber during fetal life has been extensively studied [17,18,19,20]. In the bovine skeletal muscle, myotube-multi-nucleated syncytium formed by the fusion of several myoblasts during myogenesis-formation occurs in three temporally distinct phases. The first generation of embryonic myoblasts proliferates and differentiates in myotubes to the surroundings of 30 days *post-conception* (dpc). They are completely differentiated around 180 dpc (end of the second trimester of gestation). A second and third generation of fetal myoblasts proliferate and differentiate in secondary myotubes between 60 and 90 dpc. At 180 dpc, almost all the myotubes have the appearance of muscle fibers. At this stage, the total number of myofibers is set. Contractile and metabolic maturation occurs during the last trimester. At the end of the gestation (280 dpc), the differentiation of muscle fiber types is nearly complete. 

However, there are few datums on the IMCT differentiation of the bovine fetus in vivo [21,22,23,24]. So, we hypothesized that the knowledge of the chronology of the differentiation of the different muscle tissues would allow the development of strategies (for example through maternal feeding) to enhance muscle growth and modify both IMCT and muscle fibers characteristics, and consequently their impact on final meat quality. Accordingly, we investigated the expression of ten ECM molecules thought to play an important role in the myogenesis in adults and its potential link to the quality of beef, at key stages of muscle fiber differentiation previously described by our groups [17]. The results of this study emphasized that the molecules studied are present since the beginning of fetal life in bovine and that they acquired the localization they will have in adults in the first two-thirds of fetal life (between 180 and 210 dpc). Furthermore, it appears that the main step of myogenesis occurs during the same period.

## 2. Materials and Methods 

This study was carried out in compliance with the French recommendations and those of the Animal Care and Use Committee of the National Institute for Agricultural Research (INRA, Institut National de la Recherche Agronomique) of Auvergne-Rhône-Alpes, France (under the slaughterhouse and experimental facilities license numbers #63 345 01 and #63 345.17, respectively), for the use of experimental animals including animal welfare, in accordance with the *Use of Vertebrates for Scientific Purposes Act 1985*.

### 2.1. Muscle Samples

Fifteen fetuses of 60 (*n* = 3), 110 (*n* = 3), 180 (*n* = 3), 210 (*n* = 3) and 260 (*n* = 3) days old were obtained by the artificial insemination of Charolais heifers using pure Charolais sperm. These stages have been chosen according to the key stages of muscle fiber differentiation previously highlighted in our laboratory in several studies cited in the review by Picard, et al. [17]. After the slaughter of pregnant heifers, *Semitendinosus* (ST) muscles were carefully dissected out of the two hind limbs from each animal. An approximate of 10 mm slices were taken at the mid-belly of one muscle, at right angles to the direction of the muscle fibers for histology and immunohistology and frozen in isopentane, cooled in liquid nitrogen. For electrophoresis, 3 fetuses per stage were used for 110, 180, 210 and 260 dpc. They were directly frozen in liquid nitrogen. Then all samples were stored at −80 °C until analyses. 

### 2.2. Transverse Sections Preparation

All transverse sections (10 µm thick) of ST muscle were realized with a cryotome MICROM HM 500 M at −25 °C.

### 2.3. Azorubine Staining

The muscle cells were stained with azorubine dye that stained the myofibrillar proteins in red. Sections (3 per animal) were fixed for 5 min with a solution of 5.7% formaldehyde and 18 mM CaCl_2_, washed in water and then dyed with 3% azorubine solution (Azorubine (CI 14410; Serva, Heidelberg, Germany) and 5% acetic acid for 45 min. Sections were washed in water and dehydrated twice for 1 min in acetone (Prolabo, Sion, Switzerland) and then twice for 1 min in Ottix (Microm, Brignais, France). Finally, the sections were mounted with cover-glass with Canada balsam (Prolabo, Sion, Switzerland). 

### 2.4. Antibodies 

Primary antibodies (polyclonal rabbit anti-bovine type I collagen (Col I) (catalog number, 20121), monoclonal mouse anti-human type IV collagen (Col IV) (catalog number, 20421), polyclonal rabbit anti-human type VI collagen (Col VI) (catalog number, 20611) (Novotec, Bron, France), monoclonal mouse anti bovine decorin (DCN) (catalog number, DS1) (DSHB, Iowa City, IA, USA), tenascin-X (Tn-X) and collagen XII and XIV (Col XII and Col XIV) (a generous gift from C. Lethias, IBCP, Lyon, France previously described by Berthod, et al. [25] and Elefteriou, et al. [26], chondroitin-4 and -6 sulfate (C4S and C6S) and versican (VCN) (a generous gift from B. Caterson, Cardiff University, Cardiff, UK, previously described by Hayes, et al. [27]) were diluted with 1% bovine serum albumin (BSA) in 1× phosphate buffer saline (PBS) (pH 7.2) to 1:40. Secondary antibody conjugated to Alexa Fluor 488 (Interchim, Montluçon, France) were diluted in 1% BSA in 1× PBS (pH 7.2) to 1:400.

### 2.5. Immunohistochemistry

The localization of ten ECM molecules (Col I, IV, VI, XII, XIV, DCN, Tn-X, C4S, C6S and VCN) was evidenced by the indirect immunofluorescence on four serial sections per animal according to the procedure of Listrat, Picard and Geay [22]. Natural collagen fluorescence on muscle sections was blocked with 50 mM ammonium chloride in 1× PBS for 10 min for all the used antibodies. For C4S and C6S antibodies, sections were enzymatically pre-treated with 0.5 U/mL chondroitinase ABC (Sigma-Aldrich, France) and 0.5 U/mL keratanase (Sigma-Aldrich, France) in 100 mM Tris acetate buffer (pH 7.4) for 1 hour at 37 °C to unmask the epitope then rinsed in a solution containing 0.1% Tween20 (Sigma-Aldrich, France) in 1× PBS. For versican, the sections were only incubated in 0.1% Tween20 (Sigma-Aldrich, France) in 1× PBS (without enzymatic pre-treatment) at room temperature. Then all sections were incubated in 1% BSA in 1× PBS for 10 min and they were reacted with the primary antibodies for 1 h at room temperature, washed in 1× PBS three times 5 min, incubated for 40 min at room temperature in the dark with the secondary antibody and washed in 1× PBS three times for 5 min. The sections were rinsed with 0.3% eriochrome-black T in 1× PBS to completely block natural collagen fluorescence and they were then mounted with cover-glass with Fluoromount (Sigma-Aldrich, Saint-Louis, MO, USA). Negative controls were performed by omitting primary antibodies in the same conditions that were previously described.

### 2.6. Western Blot Analyses of Type XII and XIV Collagens

Total protein extraction was performed with RIPA (Radio-Immunoprecipitation Assay) lysis buffer (150 mM NaCl, 10 mM Tris-HCl, 1 mM EGTA, 1 mM EDTA, adjusted at pH = 7.4 and completed with 100 mM sodium fluoride, 4 mM sodium pyrophosphate and 2 mM orthovanadate, 1% Triton 100×, 0.5% Igepal CA-630 and protease inhibitor cocktail (Complete, Roche Diagnostics GmbH, ref. 11 836 145 001)). After extraction, the protein concentration was determined by spectrophotometry (UVIKON 860) following the Bradford assay [28]. 

Proteins were separated in denaturing conditions (10% sodium dodecyl sulfate-polyacrylamide and β-mercaptoethanol). Samples were loaded on the gel (stacking gels of 4% and separation gels of 6%) at the rate of 50 µg of protein. Gels were run at 80 V for 20 min and then 120 V for 1 h at +4 °C.

Bands were transferred to a polyvinylidene difluoride (PVDF) membrane (ref. IPVH00010, Millipore, Burlington, MA, USA) at 120 mA for 5 h at +4 °C. The unspecific binding of antibodies to the membranes was blocked with 10% skimmed milk (Régilait) in 1× T-TBS (20 mM Tris base; 137 mM NaCl; 0.05 % Tween 20, pH 8) at 37 °C for 20 min. Membranes were washed 3 × 5 min in 1× T-TBS and then they were incubated overnight at +4 °C with the primary antibodies (anti-type XII or anti-type XIV collagen (the same than for immunohistochemistry) diluted to 1/50 in 1% skimmed milk (Régilait) in 1× T-TBS). Membranes were washed 2 × 10 min in 1× T-TBS and hybridized with the secondary antibody (diluted to 1/5000 in 1% skimmed milk (Régilait) in 1× T-TBS) associated with horseradish peroxidase for chemiluminescence detection (IgG sheep anti-mouse, NA931, GE Healthcare Life Sciences, Grenoble, France). 

Each collagen type presented three specific bands that were considered and quantified as a single band under Image Quant software (GE Health Care life Science, Grenoble, France). One volume of total protein extract of each of 15 fetuses was mixed and 50 µg of this mixed were deposited on all the gels for normalization. Each collagen type presented two or three specific bands which were considered and quantified as a single band under Image Quant software. The value obtained for the three bands of each sample was divided by the value obtained for the three bands of the mix of samples loaded on each gel. Each sample was measured in triplicate and results were expressed in arbitrary units. The other molecules were not analyzed by Western blot for technical reasons.

### 2.7. Image Acquisition and Analysis

Histological sections were visualized under an Olympus fluorescence microscope BX 51 using a 10×, 20× or 40× objective and for fluorescence an adequate band pass filter (Alexa 488: excitation filter 460–495, emission filter 510–550, dichromatic mirror 505LP). High-resolution grayscale images were acquired with an Olympus cooled digital camera DP-72 with cell-F software (Olympus Soft Imaging Solutions, Münster, Germany). Azorubine died-sections were analyzed with a VISILOG 6.7 professional software (Noesis, Gif sur Yvette, France) to calculate the mean cross-section area of fibers according to Meunier, et al. [29].

### 2.8. Statistical Analysis

The differences of relative amounts of Col XII or XIV between stages post-conception were assessed by analysis of variance (ANOVA) using the GLM procedure of SAS 9.2 Software (Statistical Analysis System, Cary, NC, USA). A probability of less than 5% was considered statistically significant. All results were presented as least square means ± Standart Error of the Mean (SEM). 

## 3. Results

### 3.1. Sixty and 110 dpc

#### 3.1.1. Perimysium

**Figure 1:**
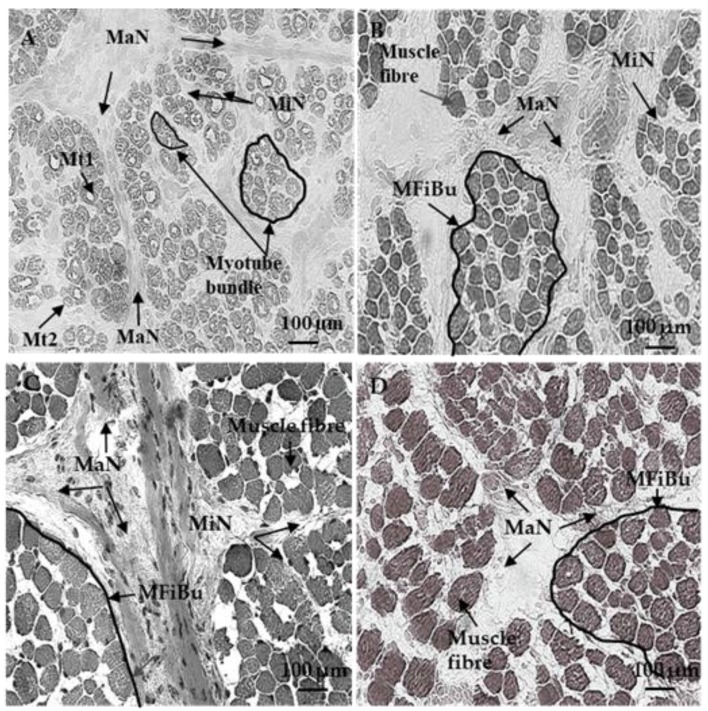
Azorubine staining of transverse sections of foetal Semitendinosus muscle at 110 (**A**), 180 (**B**), 210 (**C**) and 260 (**D**) days post-conception (dpc). (ECM: ExtraCellular Matrix; MaN: perimysium major network, MiN: perimysium minor netork, Mt1: primary myotubes, Mt2: secondary myotube, MFiBu: Muscle Fiber Bundle).

**Figure 2:**
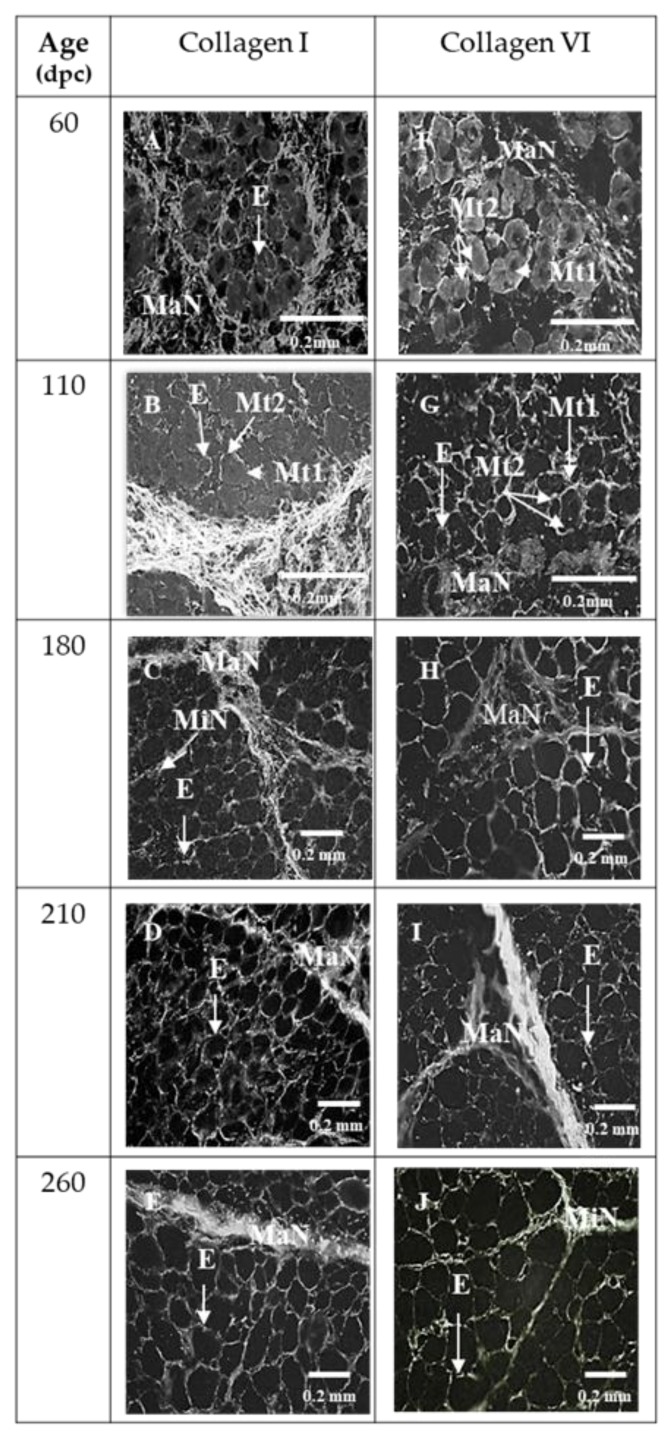
Immunohistochemical labelling with antibodies against type I (**A**, **B**, **C**, **D**, **E**), and type VI (**F**, **G**, **H**, **I**, **J**) collagens of transverse sections of foetal Semitendinosus muscle at 60, 110, 180, 210 and 260 days post-conception (dpc) (Major (MaN) and minor (MiN) networks of perimysium; E: endomysium, Mt1: primary myotubes, Mt2: secondary myotubes).

**Figure 3:**
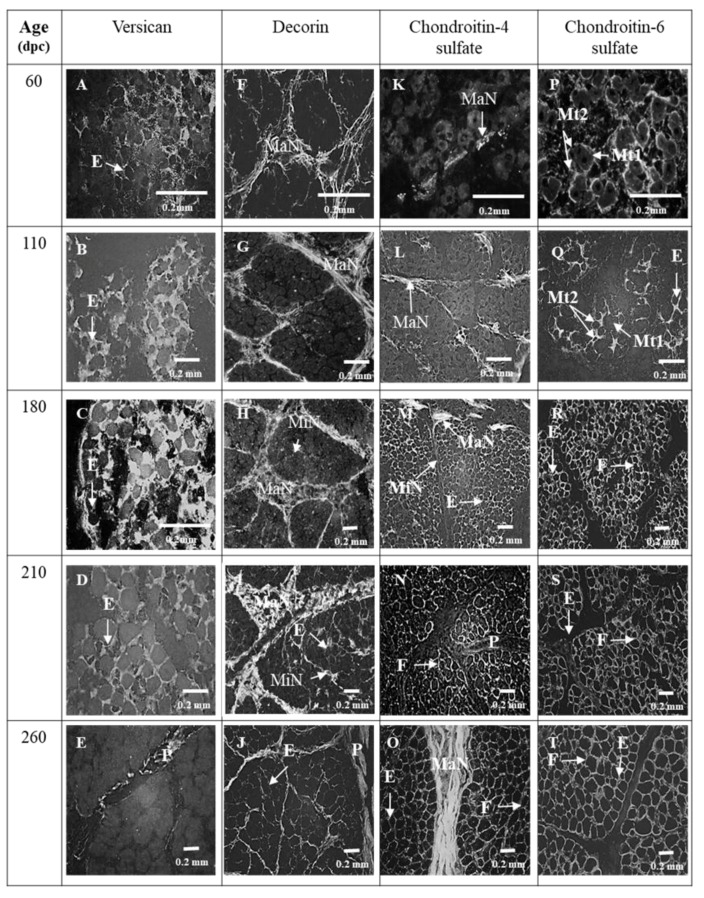
Immunohistochemical labelling with antibodies against versican (**A**, **B**, **C**, **D**, **E**), decorin (**F**, **G**, **H**, **I**, **J**), chondroitin-4-sulfate (**K**, **L**, **M**, **N**, **O**), chondroitin-6-sulfate (**P**, **Q**, **R**, **S**, **T**) of transverse sections of foetal Semitendinosus muscle at 60, 110, 180, 210 and 260 days post-conception (dpc). (Major (MaN) and minor (MiN) networks of perimysium (P), E: endomysium, Mt1: primary myotubes, Mt2: secondary myotubes, F: muscle fiber).

**Figure 4:**
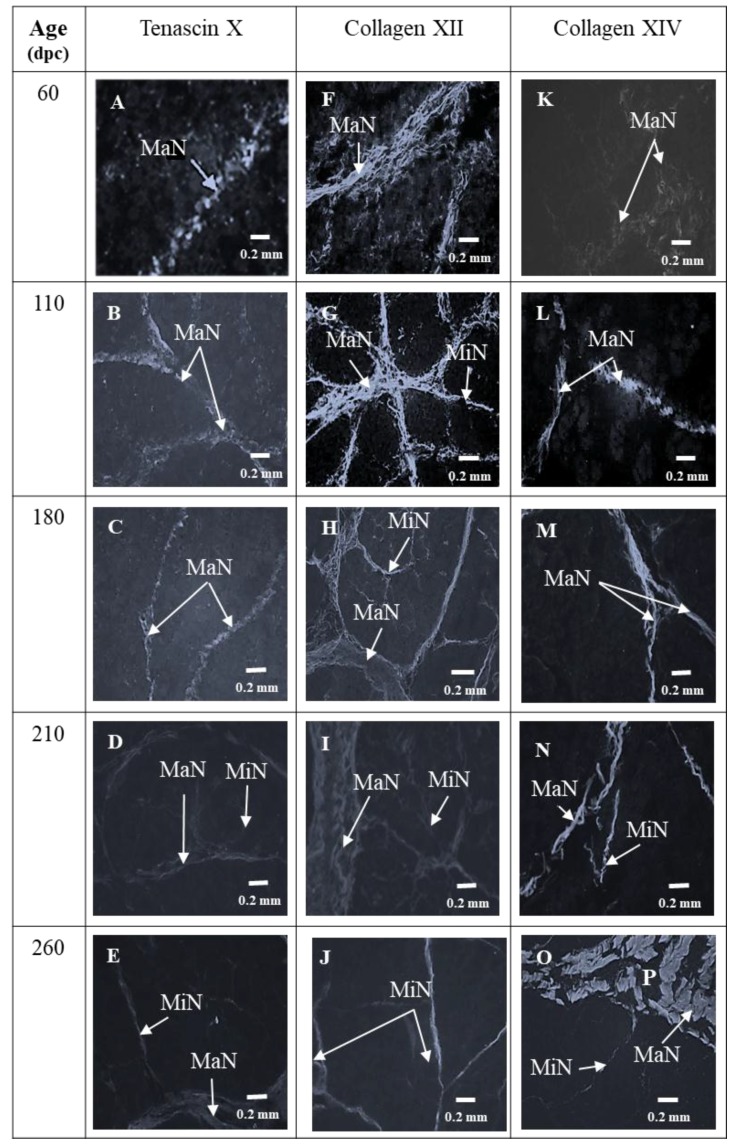
Mmunohistochemical labelling with antibodies against tenascin-X (**A**, **B**, **C**, **D**, **E**), collagen XII (**F**, **G**, **H**, **I**, **J**), collagen XIV (**K**, **L**, **M**, **N**, **O**) of transverse sections of foetal Semitendinosus muscle at 60, 110, 180, 210 and 260 days post-conception (dpc). (Major (MaN) and minor (MiN) networks of perimysium).

At 60 and 110 dpc, myotubes were grouped in bundles individualized by a wide perimysium divided into a major (around the muscle cell bundles) and minor network (around and inside muscle cell bundles) (Figure 1A). The average area of the myotubes was 84.2 ± 15.9 µm^2^ at 60 dpc and 85.5 ± 17.5 µm^2^ at 110 dpc. At 60 and 110 dpc, future perimysium was stained by Col I (Figure 2A,B), VI (Figure 2F,G), DCN (Figure 3F,G), C4S (Figure 3K,L), Tn-X (Figure 4A,B) and Col XII (Figure 4F,G). At 60 dpc, Col XIV was undetectable (Figure 4K). At this stage, C4S (Figure 3K) and TN-X (Figure 4A) labeling were very low. At 110 dpc, the presence of C4S, Tn-X (Figure 3L and Figure 4B), Col XIV (Figure 4L) in the perimysium was confirmed, but only in major networks.

At 60 dpc, Col XII was present in major networks of perimysium (Figure 4F) while at 110 dpc (Figure 4G), it was present everywhere in the perimysium. 

**Figure 5:**
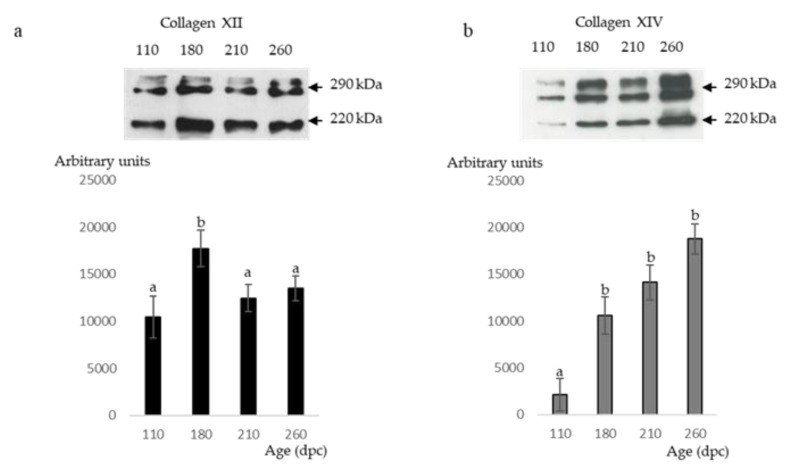
(**a**) Collagen XII and (**b**) Collagen XIV Western blot analysis of foetal Semitendinosus muscle at 110, 180, 210 and 260 days post-conception (dpc). The monoclonal antibodies against collagen XII and XIV, two disulphide bonded polypeptides, recognized three bands on Western blots of bovine muscle extracts, one band at 220 kDa and two others at about 290 kDa. The presence of these three bands was due to the fact that migration conditions were reducing. The relative amounts of collagen XII and XIV (least square means ± standard error of the mean) were expressed in arbitrary units. Different letters on the same graph indicated that relative amounts of collagen XII and XIV differed significantly between foetal stages (*p* < 0.05).

At all studied stages (110, 180, 210 and 260 dpc), the presence of both Col XII and XIV were observed as three bands of molecular weight between 220 and 290 kDa (Figure 5a,b).

#### 3.1.2. Endomysium

**Figure 6:**
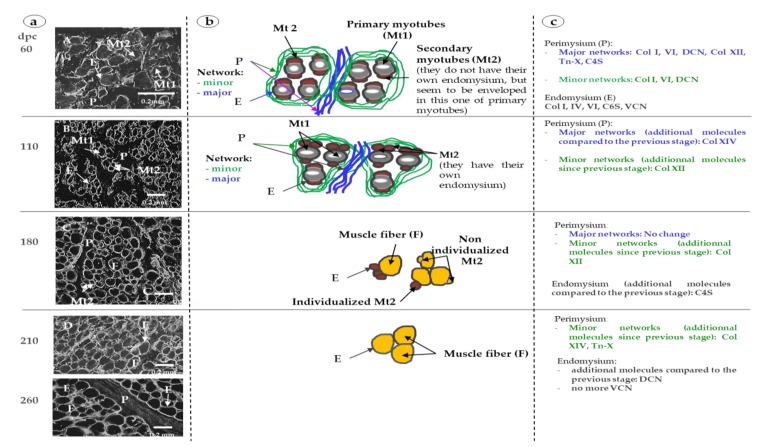
(**a**) The immunohistochemical labeling with antibodies directed against collagen IV (**A**, **B**, **C**, **D**, **E**) of transverse sections of fetal *Semitendinosus* muscle at 60, 110, 180, 210 and 260 days *post-conception* (dpc). (P: perimysium, E: endomysium, Mt1: primary myotubes, Mt2: secondary myotubes, F: muscle fiber). (**b**) Schematic representation of differentiation of muscle fibers at 60, 110, 180, 210 and 260 days post-conception (dpc). At 60 and 110 dpc, muscle fibers are present as primary (cells schematically represented by an oval grey form with a central lumen surrounded by a dark grey line representing endomysium) myotubes (Mt1) and secondary myotubes (Mt2) (cells schematically represented by a brown form without central lumen). At 60 dpc, Mt2 are not individualized; they are wrapped in the same endomysium than Mt1. At 110 dpc, almost all Mt2 are individualized and they have their own endomysium (dark grey line). At 180 and 210 dpc, some Mt2, individualized or not, are still present, but the majority of muscle cells are fibers (schematically represented by a yellow form surrounded by a dark grey line representing endomysium). At 260 dpc, all myotubes are muscle fibers. (**c**) Schematic description of localization of different molecules of ExtraCellular matrix. At each stage, the localization of ECM molecules (Col I, IV, VI, XII and XIV: collagen of type I, IV, VI, XII and XIV; DCN: decorin, TN-X: tenascin-X, C4 and 6S: chondroitin 4 and 6 sulfate; VCN: versican) is indicated in the major and minor networks of perimysium and in the endomysium. From 110 dpc, only the modifications of localization of ECM molecules are indicated.

At 60 and 110 dpc, endomysium was distinctly labeled by Col I, VI (Figure 2A,B; Figure 2F,G), IV (Figure 6A,B) and C6S (Figure 3P,Q). At these stages, labeling obtained with an antibody against VCN was diffuse and occupied all the space between myotubes (Figure 3A,B). C4S appeared in endomysium between 60 and 110 dpc (Figure 3K,L).

For VCN, C6S (Figure 3A,B and Figure 3P,Q, respectively), Col VI and IV (Figure 2F,G and Figure 6A,B), the main difference between 60 and 110 dpc was the individualization or non-individualization of primary and secondary myotubes. At 60 dpc, secondary and primary myotubes were not individualized from each other, but seemed wrapped in the same endomysium in bundles of 2 or 3 myotubes while at 110 dpc, some secondary myotubes began to be well individualized from primary myotubes, while others always seemed wrapped in the same endomysium. This result is illustrated in Figure 6 by both labeling with the antibody anti-Col IV (Figure 6a) and with a diagram (Figure 6b).

### 3.2. 180 dpc to 260 dpc

At 180 dpc, almost all muscle cells had the appearance of muscle fibers and were organized into bundles as in adults (Figure 1B). The average area of muscle fibers (138.2 ± 34.5 µm^2^) was significantly higher (*p* < 0.05) than that at 110 dpc. The fiber cross-section area significantly (*p* < 0.05) and progressively increased (282.53 ± 74.5 µm^2^ at 210 dpc to 391.22 ± 116.1 µm^2^ at 260 dpc) between 180 and 260 days.

#### 3.2.1. ECM Molecules Whose Location Changed

C4S (Figure 3M,N), Tn-X and Col XIV (Figure 4C,D and Figure 4M,N) began to appear in minor networks of perimysium between 180 and 210 dpc. The measures of Col XII and XIV by Western blotting showed that the relative amount of Col XII significantly increased between 110 dpc and 180 dpc then significantly decreased between 180 and 260 dpc (Figure 5a). The relative amounts of Col XIV significantly increased between 110 and 260 dpc (Figure 5b). 

DCN appeared in the endomysium and in the minor networks of perimysium between 180 and 210 dpc (Figure 3H,I). C4S appeared in the minor networks of the perimysium between 110 and 180 dpc (Figure 3L,M). VCN was present in the endomysium until 210 dpc (Figure 3D) then disappeared from the endomysium between 210 and 260 dpc (Figure 3D,E). At 260 dpc, it was only expressed in the perimysium (Figure 3E). 

#### 3.2.2. ECM Molecules Whose Location Did not Change

From 180 dpc, the localization of Col I, VI (Figure 2C,H), IV (Figure 6C), and of C6S (Figure 3R) did not change anymore. Col I and VI were present both in the endo- and perimysium, Col IV and C6S only in endomysium. As at 110 dpc, Col XII was present in the entire network until 260 dpc (Figure 4H,I,J). All modifications of the localization of molecules of ECM were summarized in Figure 6c.

## 4. Discussion

This report described the chronology of differentiation of 10 ECM molecules in the bovine fetus. According to their chronology of differentiation and their localization, the molecules can be classified into several groups. The molecules (i) exclusively located in endomysium (Col IV, C6S), those (ii) exclusively located in perimysium (Col XII and XIV, TN-X), those (iii) first located in perimysium then appearing in endomysium more or less precociously (Col I, VI, DCN and C4S) or (iv) such as VCN first located in endomysium, then (v) that disappeared and appeared in perimysium at the end of the fetal life. 

In this study, Col IV was the collagen type detected the earliest in endomysium (since 60 dpc). It remained present in endomysium throughout fetal life as well as in adults. Pöschl, et al. [30] observed that embryos developed up to E9.5 on a null allele of the Col 4a1/2 locus in mice. However, lethality occurred between E10.5–E11.5 (about 30 dpc for the bovine) because of structural deficiencies in the basement membranes. The data of these authors highlighted that Col IV is fundamental for the maintenance of the integrity and function of basement membranes and, consequently, must be present very early in the process of differentiation.

As previously shown, Col I and VI were present since 60 dpc in the perimysium [23,31]. Col VI appeared progressively in the endomysium of primary and secondary myotubes between 60 and 110 dpc; it was colocalized with Col I. At 180 dpc, Col I and VI had the localization that they will have in the adult muscle in the endomysium and the perimysium. The period between 60 and 110 dpc coincides with the differentiation of fetal myoblasts in myotubes. Thus, as previously suggested in vivo by Listrat, Picard and Geay [22] and Listrat, Picard and Geay [23] and in vitro by several other authors [32,33], our results suggest that Col I and VI might be involved in the early differentiation of myotubes. However, recent studies support the idea that these collagens do not play a direct role in differentiation. Their presence would enable the preservation of collagen-binding molecules like proteoglycans, laminins and fibronectin and, consequently, the cytokines and growth factors that bind these molecules and are essential for myoblast differentiation in myotubes [34]. 

In chicken, VCN is transiently upregulated in myoblasts and newly formed myotubes. It is located in the endomysium. At the same stages and from chicken, decorin is observed in the perimysium, then its distribution gradually spreads to the perimysium and endomysium at the end of ovogenesis [35,36]. These observations are in line with what we observed in this study in bovine fetuses since VCN was present in the endomysium between 60 and 210 dpc, disappeared and were then replaced by DCN between 210 and 260 dpc. In bovine fetuses, the proliferation and differentiation of myoblasts in myotubes are at their highest peak between 60 and 210 dpc [37]; stages of the presence of VCN in endomysium. The role of VSC in the myogenesis is poorly defined, even though it may be an important driver during myogenesis [38]. However, it is known that it contributes to the formation of a hydrated pericellular matrix—part of ECM in close contact with cells—whose remodeling by some proteinases of ADAMTS family (a disintegrin and metalloproteinase with thrombospondin motifs) contributes to the formation of myotubes [38]. 

At 210 dpc, the total number of muscle fibers is fixed, the contractile differentiation of the first generation of myotubes is completed, and various adult myosin heavy chain (MHC) isoforms begin their expression [37]. We suggest that DCN could have an indirect but crucial role at this stage. Indeed, Miura, et al. [39] proposed that DCN would decrease the activity of myostatin—a growth factor—by its sequestration. A lack of myostatin [40] and an over-expression of DCN [41] would lead to the upregulation of MyoD (myoblast determination protein) and reduce the levels and activity of MEF2 (myocyte enhancer factor-2)—a transcription factor—and calcineurin—a protein phosphatase. MyoD is a transcription factor of the myogenic factors subfamily involved in muscle differentiation which is important for the activation of MHC IIB gene expression [41]. The loss of MEF2 and calcineurin may result in a reduction of slow fibers [42]. Thus, when decorin began to be present in endomysium, it could begin to regulate the fiber-type composition of skeletal muscles by regulating MEF2, calcineurin and MyoD gene expression. 

The results of this study showed that, between 110 and 180 dpc, the relative amounts of Col XII increased then, after 180 dpc, it decreased. This decrease could be involved in muscle fiber type transitions that occur at the end of fetal life and, more precisely, in the differentiation of type II fibers as suggested by the work of Zou, et al. [43] in the model of mouse Col12A1^−/−^. In this model, in two muscles, the *Soleus* (slow oxidative) and the *Tibialis* (fast glycolytic), the absence of Col XII results in a more glycolytic metabolism. Nishiyama, McDonough, Bruns and Burgeson [14] have shown that the addition of this collagen type to collagen gels modifies their mechanical properties, suggesting that Col XII might be directly involved in determining the mechanical properties of collagen-rich tissues in vivo and their elasticity. Consequently, it has been proposed that the reduction of relative quantities of Col XII could change the matrix elasticity and thereby facilitate the terminal differentiation of muscle fibers [43].

In our model, the decrease of the relative amounts of collagen XII was associated with an increase of Col XIV. At the same period (between 180 and 210 dpc), Tn-X, which was already present in major perimysium networks, appeared in minor ones. To our knowledge, it is not known whether Tn-X or Col XIV play a role in the regulation of myogenesis. However, when Tn-X [13] and Col XIV [14] are added to collagen gels, they modify their mechanical properties and could act in synergy with Col XII. 

## 5. Conclusions

This study is the first one to investigate the chronology of appearance of 10 ECM molecules of several families (collagens, PGs, …) in bovines along the fetal life. The main factor investigated is the age of fetuses at key stages of myogenesis as previously defined in our laboratory [38]. This approach highlighted, for the first time, that since 60 dpc, all the studied ECM molecules were present. They acquired the localization they will have in adults in the first two-thirds of fetal life (between 180 and 210 dpc). The main step of myogenesis terminates during the same period. Around 180 dpc, the number of muscle fibers is fixed and adult MHC forms begin to be present. The findings of this study also suggest that to consider controlling the muscular differentiation, it would be necessary to act very early (before the ECM constituents have acquired their definitive localization and that the muscle fibers have finished differentiating) at the beginning of the first third of gestation. The knowledge of key stages of differentiation of different tissues of muscle is essential in order to be able to control the characteristics in relation to beef quality, for example, through maternal feeding. Indeed, in ruminants, it is known that it is possible to decrease the number of secondary myotubes through the nutrient deficiency of the dam from early-to-mid gestation and during mid-to-late gestation to decrease the number of intramuscular adipocytes and muscle fiber sizes [44]. The role of maternal feeding on IMCT is less known, but Huang, et al. [45] have shown that maternal over-nutrition increased collagen accumulation and cross-linking in skeletal muscle offspring.
